# Supplementary material for: Comparison of Liquid-Based Preparations with Conventional Smears in Thyroid Fine-Needle Aspirates: A Systematic Review and Meta-Analysis
Source: Cancers (Basel). 2024 Feb 11;16(4):751. doi: 10.3390/cancers16040751 (PMC10886914; doi:10.3390/cancers16040751)
Supplement: Supplementary file 1 [file cancers-16-00751-s001.zip › cancers-2832838-supplementary.pdf]

Supplementary Table S1. Diagnostic subjects and excluded intermediate case of the included studies.

Study	Diagnostic subject	Intermediate case [a]
Scurry 2000	Not defined	17 lesions
Scurry 2000	Not defined	17 lesions
Afify 2001	Not defined	6 lesions
Afify 2001	Not defined	9 lesions
Cochand-Priollet 2003	Per specimen	3 lesions
Cochand-Priollet 2003	Per specimen	2 lesions
Kim 2007	Per specimen	12 lesions
Kim 2007	Per specimen	14 lesions
Cavaliere 2008	Per specimen	24 lesions
Cavaliere 2008	Per specimen	17 lesions
Jung 2008	Per specimen	0 lesion
Jung 2008	Per specimen	0 lesion
Luu 2010	Per specimen	45 lesions
Luu 2010	Per specimen	44 lesions
Koo 2011	Per specimen	20 lesions
Koo 2011	Per specimen	13 lesions
Kim 2011	Per specimen	11 lesions
Kim 2011	Per specimen	12 lesions
Chang 2012	Per specimen	57 lesions
Chang 2012	Per specimen	34 lesions
Nagarajan 2015	Per specimen	340 lesions
Nagarajan 2015	Per specimen	20 lesions
Kumari 2020	Per specimen	2 lesions
Kumari 2020	Per specimen	1 lesion
Rufail 2020	Per person	9 lesions
Rufail 2020	Per person	35 lesions
Zhao 2020	Per person	33 lesions
Zhao 2020	Per person	11 lesions
Ucak 2021	Per person	219 lesions
Ucak 2021	Per person	220 lesions
Ucak 2021	Per person	215 lesions
Maurya 2023	Per person	17 lesions
Maurya 2023	Per person	18 lesions
Xiong 2023	Not defined	33 lesions
Xiong 2023	Not defined	6 lesions

a: Atypia of undetermined significance (Bethesda III) or suspicious for follicular neoplasm (Bethesda IV))

Supplementary Table S2. Comparative analysis of sensitivities, specificities, negative predictive values, diagnostic odd ratios, and rate of inadequate specimen.

	Diagnostic odd ratios	Sensitivity	Specificity	Negative predictive values	Area under the summary receiver operating characteristic curve	Rate of inadequate specimen
Conventional smear	N=15 23.6674 [13.4718; 41.5794]; I^2=82.6%	N=15 0.8266 [0.7498; 0.8835]; I^2=87.2%	N=15 0.8668 [0.7721; 0.9259]; I^2=96.1%	N=15 0.8969 [0.7805; 0.9552]; I^2=97.6%	N=15 0.879	N=19 0.1280 [0.0865; 0.1853]; I^2=98.5%
Liquid-based preparation	N=16 25.3587 [7.1871; 89.4747]; I^2=95.9%	N=16 0.8190 [0.7459; 0.8746]; I^2=83.8%	N=16 0.8833 [0.7348; 0.9539]; I^2=97.8%	N=16 0.8515 [0.7124; 0.9300]; I^2=95.6%	N=16 0.865	N=16 0.1729 [0.1231; 0.2375]; I^2=97.0%
Combining conventional smear and liquid-based preparation	N=4 9.4557 [3.2976; 27.1139]; I^2=93.6%	N=4 0.7809 [0.6976; 0.8463]; I^2=79.6%	N=4 0.7267 [0.5370; 0.8591]; I^2=95.8%	N=4 0.8111 [0.6388; 0.9125]; I^2=95.2%	N=4 0.813	N=3 0.1109 [0.0901; 0.1357]; I^2=82.1%
P value	0.2979	0.6232	0.1743	0.5432	0.795	0.0876

Supplementary Table S3. Subgroup analysis of sensitivities, specificities, negative predictive values, diagnostic odd ratios, and rate of inadequate specimen.

	Diagnostic odd ratios	Sensitivity	Specificity	Negative predictive values	Area under the summary receiver operating characteristic curve
All<br>N=22	26.9769 [8.9211; 81.5767]; I^2=94.7%	0.8334 [0.7678; 0.8833]; I^2=82.7%	0.8637 [0.7481; 0.9312]; I^2=96.5%	0.8769 [0.7522; 0.9435]; I^2=96.0%	0.871
Conventional smear<br>N=10	32.1250 [10.6191; 97.1846]; I^2=85.5%	0.8487 [0.7411; 0.9167]; I^2=78.4%	0.8659 [0.7408; 0.9359]; I^2=92.8%	0.9107 [0.7408; 0.9733]; I^2=94.8%	0.898
Liquid-based preparation (SurePath)<br>N=7	19.7734 [1.6023; 244.0203]; I^2=93.5%	0.8573 [0.6806; 0.9442]; I^2=86.4%	0.8368 [0.4211; 0.9731]; I^2=94.6%	0.7573 [0.5036; 0.9057]; I^2=87.4%	0.841
Liquid-based preparation (Thin-Prep)<br>N=5	29.1494 [4.9108; 173.0254]; I^2=89.6%	0.8182 [0.7403; 0.8767]; I^2=8.7%	0.9080 [0.7064; 0.9759]; I^2=94.7%	0.8988 [0.5741; 0.9832]; I^2=96.4%	0.791
P value	0.9416	0.8051	0.8346	0.3463	0.061
